# Activation of β-catenin and Akt pathways by Twist are critical for the maintenance of EMT associated cancer stem cell-like characters

**DOI:** 10.1186/1471-2407-11-49

**Published:** 2011-02-01

**Authors:** Junlin Li, Binhua P Zhou

**Affiliations:** 1 Departments of Molecular and Cellular Biochemistry, University of Kentucky School of Medicine, Lexington, KY 40506, USA; 2 Markey Cancer Center, University of Kentucky School of Medicine, Lexington, KY 40506, USA

## Abstract

**Background:**

Epithelial-mesenchymal transition (EMT) not only confers tumor cells with a distinct advantage for metastatic dissemination, but also it provides those cells with cancer stem cell-like characters for proliferation and drug resistance. However, the molecular mechanism for maintenance of these stem cell-like traits remains unclear.

**Methods:**

In this study, we induced EMT in breast cancer MCF7 and cervical cancer Hela cells with expression of Twist, a key transcriptional factor of EMT. The morphological changes associated with EMT were analyzed by immunofluorescent staining and Western blotting. The stem cell-like traits associated with EMT were determined by tumorsphere-formation and expression of ALDH1 and CD44 in these cells. The activation of β-catenin and Akt pathways was examined by Western blotting and luciferase assays.

**Results:**

We found that expression of Twist induced a morphological change associated with EMT. We also found that the cancer stem cell-like traits, such as tumorsphere formation, expression of ALDH1 and CD44, were significantly elevated in Twist-overexpressing cells. Interestingly, we showed that β-catenin and Akt pathways were activated in these Twist-overexpressing cells. Activation of β-catenin correlated with the expression of CD44. Knockdown of β-catenin expression and inhibition of the Akt pathway greatly suppressed the expression of CD44.

**Conclusions:**

Our results indicate that activation of β-catenin and Akt pathways are required for the sustention of EMT-associated stem cell-like traits.

## Background

Tumor recurrence is one of the biggest challenges in breast cancer, because it often leads to an incurable disease. Therapeutic resistance, the major mechanism underlying tumor recurrence, raises the question of whether conventional anticancer therapies target the correct cells. The existence of a subpopulation of tumor cells with stem cell-like characteristics, such as very slow replication and resistance to standard chemotherapy, poses a new concept to account for the phenomena of drug resistance and tumor recurrence. It was not until 1994 that cancer stem cells (CSCs, also known as tumor-initiating cells) were first identified in human acute myeloid leukemia malignancies [1]. Subsequent studies have identified CSCs in solid tumors, including breast [2], prostate [3,4], brain [5], colon [6], and pancreas [7,8]. For example, breast cancer stem cells are characterized by low levels of heat stable antigen (CD24) and high levels of hyaluronan receptor (CD44) expression. This subpopulation of cells has the ability to self-renew, and to initiate tumor formation, and is intrinsically resistant to therapy. The cancer stem cell hypothesis has fundamental clinical implications, as current treatment strategies may affect the bulk of the tumor cells but leave CSCs behind, serving as a reservoir for disease recurrence and metastasis [9-11]. Therefore, the elucidation of molecular pathways, which regulate self-renewal activity of CSCs and their interaction with niche, will provide potential therapeutic targets.

Although the CSCs hypothesis suggests that tumors can arise from stem or progenitor cells, studies from many laboratories indicate that epithelial-mesenchymal transition (EMT) can endow cells with stem-cell like characteristics [12-15]. EMT is an embryonic developmental process in which epithelial cells lose expression of many markers of differentiation, acquire fibroblast-like properties and show reduced intercellular adhesion and increased motility [16-18]. EMT has been recognized not only as a physiological mechanism for development and tissue remodeling, but also as a pathological mechanism in the progression of various diseases including inflammation, fibrosis and cancer [16,17]. Weinberg and his colleagues showed that induction of EMT in immortalized human mammary epithelial cells results in an increased ability to form tumorspheres, and in the expression of stem cell-like markers [13]. Specifically, cells with CD44+CD24low phenotype, which yielded tumor formation with as few as 100 cells (compared with that the control), were found significant increased when cells were treated with transforming growth factor-beta or were overexpressing the key EMT inducers, Snail and Twist. These data indicate that EMT endows tumor cells with stem cell-like properties. Consistent with this finding, tumor cells resistant to chemo- and endocrine therapies activate the EMT program, which results in the expansion of CSCs with CD44^+^CD24^low ^expression [13,14,19]. However, it is unclear how the activation of the EMT program contributes to the expansion of CSCs with CD44^+^CD24^low ^traits.

A hallmark of EMT is the loss of *E-cadherin *expression [16-18]. E-cadherin is a cell-cell adhesion molecule that participates in homotypic, calcium-dependent interactions to form epithelial adherent junctions [20,21]. Loss of E-cadherin expression is often correlated with the tumor grade and stage [20,21], because it results in the disruption of cell-cell adhesion and an increase in nuclear β-catenin, thus leading to cell growth and survival. On one hand, β-catenin is an essential component of adherent junctions, where it provides the link between E-cadherin and β-catenin and modulates cell-cell adhesion and cell migration [22]. On the other hand, β-catenin also functions as a transcription cofactor with T cell factor (TCF). In unstimulated cells, the level of free cytoplasmic β-catenin is kept low through a destruction complex, which consists of axin, adenomatous polyposis coli (APC), GSK-3β and casein kinase (CKI). GSK-3β phosphorylates β-catenin and triggers its ubiquitination and degradation by β-Trcp. In the presence of Wnt ligands, Wnts bind to frizzled and LRP5/6 receptor complex to inactivate GSK-3β in the destruction complex. This, in turn, results in the stabilization and nuclear accumulation of β-catenin and leads to the activation of the Wnt/β-catenin signaling pathway [22], which has been implicated in stem cell maintenance and self-renewal.

In this study, we found that the expression of Twist induced EMT and the expansion of the CD44^high^CD24^low ^subpopulation, which is associated with CSC properties. We showed that β-catenin and Akt pathways were activated in these Twist-overexpressing transfectants. The nuclear accumulation of β-catenin correlated with the expression of CD44. Knockdown of β-catenin expression and inhibition of the Akt pathway significantly decreased the expression of CD44. Together, our results indicate that the activation of β-catenin and the Akt pathway is required for the sustention of cancer stem cell-like traits generated by EMT.

## Methods

### Cell cultures, transfections and reporter assays

MCF7 and Hela cells were cultured with DMEM medium supplemented with 10% fetal bovine serum in a humidified CO_2 _incubator at 37°C. To generate Twist-expression stable transfectants, Hela and MCF7 cells were transfected with pcDNA3-Twist1, and stable clones were selected with 1000 μg/ml of G418 (CALBIOCHEM) for 4 weeks.

TOPflash or FOPflash plasmid (Upstate, Lake Placid, NY) was transiently transfected into cells with Fugene 6 (Roche, Indianapolis, IN). For measuring the transcription of CD44, pGL3-CD44P was also expressed in cells. To normalize transfection efficiency, cells were also co-transfected with 0.1 μg of the pRL-CMV (Renilla luciferase). Forty-eight hours after transfection, luciferase activity was measured using the Dual-Luciferase Assay kit (Promega, Madison, WI). Three independent experiments were performed, and the calculated means and standard deviations are presented.

To knock down the expression of β-catenin, cells were seeded on 6-well plates and transfected with pGL3-CD44P, along with validated human β-catenin siRNA (Dharmacon) at a final concentration of 100 nM using X-tremeGENE siRNA transfection reagent (Roche) following manufacturer's instructions. After 36 h of transfection, cells were treated with or without PI3K/Akt inhibitors wortmannin (100 nM) for overnight. Luciferase activity was measured as described above. All experiments were performed at least three times in triplicate.

Commercial antibodies used in this study were presented in Table 1.

**Table 1:** Antibodies used in this study

Name	Antigen	Source	Manufacturer
Twist	Twist	Rabbit	Cell signaling
Snail	Snail	Rat	Cell signaling
N-cadherin	N-cadherin	Mouse	Upstate
Fibronectin Ab-11	Fibronectin	Mouse	Neo Markers
Vimentin Ab-2	Vimentin	Mouse	Neo Markers
γ-catenin	γ-catenin	Mouse	BD transduction
E-cadherin	E-cadherin	Mouse	BD transduction
ZO-1	ZO-1	Rabbit	Abcam
p-GSK-3β (Ser9)	GSK-3β (Ser9)	Mouse	Upstate
GSK-3β	GSK-3β	Goat	Santa Cruz
p-Akt (Ser473)	Akt (Ser473)	Rabbit	Cell signaling
Akt	Akt1,2,3	Rabbit	Cell signaling
p-β-catenin(Ser33/37/Thr41)	β-catenin (Ser33/37/Thr41)	Rabbit	Cell signaling
β-catenin	β-catenin	Rabbit	Abcam
β-tubulin	β-tubulin	Mouse	Sigma
Anti-FLAG M2	FLAG-tag	Mouse	Sigma
CD44	CD44	Mouse	Cell signaling
PE-Cy7-CD44	CD44	Rat	eBioscience
PE-CD24	CD24	Mouse	eBioscience

### Western Blot Analysis

To prepare the whole-cell extract, cells were washed with PBS once and harvested by scraping them in 1 ml lyses buffer (50 mM Tris-HCl pH7.4, 150 mM NaCl, 0.2 mM EDTA, 0.2% NP-40, 10% Glycerin, 1M β-Me, 1 μg/ml Aprotin, 0.5 μg/ml Leupetin, 0.1 mM Na_3_VO_4_, 0.5 mM 4NPP, 0.5 mM NaF, and protease inhibitors). Cellular lysates were centrifuged at 13,200 × g for 5 min at 4°C. Protein content was determined by the Bradford assay (Bio-Rad Laboratories, Hercules, CA). The extracted proteins were separated in a 10-12% SDS-polyacrylamide gel electrophoresis and transferred to a nitrocellulose membrane (Amersham Bioscience). The membranes were first blocked with 5% (w/v) nonfat dry milk in PBST and then probed with the indicated primary antibodies with gentle shaking at 4°C overnight. After washing the membranes four times, the membranes were incubated with the appropriate peroxidase-conjugated secondary antibodies for 1 hour. The signals were detected using an enhanced chemiluminescence kit (Amersham Biosciences).

### Immunofluorescent Analysis

Cells were grown on glass chamber slides fixed with 4% paraformaldehyde in PBS for 30 min. Then cells were permeabilized in 0.1% Triton X-100 for 30 min and blocked with 0.5% bovine serum albumin in PBS for 30 min at room temperature. After washing with PBS, the cells were incubated with specific primary antibodies for 1 hour at room temperature. After being washed with PBST, the cells were incubated with appropriate fluorescein isothiocyanate-conjugated secondary antibodies and then stained with 4', 6-diamidino-2-phenylindole (DAPI) (Roche Diagnostics). The images were visualized with an Olympus microscope.

### Flow Cytometry Analysis

Flow Cytometry Analysis was performed as described previously [23]. Cells were harvested by trypsinization and washed twice with PBS. The cells then were fixed and stained with monoclonal antibodies against CD44, CD24 or an isotype IgG, labeled with Alexa 488-conjugated secondary antibody, and subjected to flow cytometric analysis using a flow cytometer (BD-LSR model, Becton-Dickinson, San Jose, CA).

### Tumorsphere Culture

Single-cell suspensions were suspended at a density of 4,000 cells per milliliter in Dulbecco's modified Eagle's medium/F-12 (Twist/MCF7, MCF7) or Dulbecco's modified Eagle's medium (Twist/HeLa; HeLa) and seeded into six-well plates (2.0 mL per plate) coated with 1.2% poly-Hema. Suspension cultures were continued for 1-2 weeks (7-day for Hela and Twist/Hela cells; 12-day for MCF7 and Twist/MCF7 cells) until the formation of tumorspheres. Colonies were counted at 10 different views under microscope. Experiments were repeated three times with duplication in each experiment.

#### Cellular Fractionation Analysis

Cellular fractionation was performed as described by Abmayr et al with minor modifications [24]. Briefly, cells were harvested with trypsinization and washed twice with phosphate-buffered saline (137 mM NaC1, 2.7 mM KC1, 4.3 mM Na_2_HPO_4_, 1.4 mM KH_2_PO_4_, pH 7.4). Cells were rapidly washed once with hypotonic buffer (10 mM HEPES, pH 7.9, 1.5 mM MgCl_2_, 10 mM KCl, 0.2 mM PMSF, and 0.5 mM DTT), re-suspended with 3 packed cell volume of hypotonic buffer and allowed to swell on ice for 10 min. Cells were then homogenized with 20 strokes on Dounce homogenizer (type B pestle) to ensure that >95% of cells were lyzed. After centrifugation at 4°C with 3300 × g for 15 min, Supernatant was saved for S-100 cytoplasmic extract preparation. The nuclear pellet was washed once with lysis buffer (50 mM Tris-HCl pH7.4, 150 mM NaCl, 0.2 mM EDTA, 0.2% NP-40, 10% Glycerin, and protease cocktail) and suspected in the same buffer. After brief sonication, the suspension was spin at 13,200 × g for 20 min and supernatant was saved as the nuclear fraction. To prepare the membrane and cytoplasmic fractions, the supernatant saved above was centrifuged at 100,000 × g for 20 minutes at 4°C, Supernatant was saved as the cytoplasmic fraction. The pellet was re-suspended in lysis buffer containing 1% of Trition X-100 and save as the membrane fraction.

Equal proteins from these three fractions for parental and Twist-overexpressing cells were used for western blotting analysis.

#### Preparation of Wnt3a Conditioned-Medium

Wnt3A-conditioned media was prepared as described by Willert et al [25]. Briefly, stable murine L-cells (ATCC, Manassas, VA) that overexpress Wnt3A were maintained in Dulbecco's modified Eagle's medium supplemented with 10% fetal bovine serum, 1% L-glutamine, and 0.4 mg/ml Geneticin. To obtain Wnt3A-conditioned media, cells were seeded into 100-mm dishes and cultured for 4 days in growth medium without G418, the medium was removed and sterile-filtered. Fresh medium was added to the plates and cultured for an additional 3 days. The medium was then removed, sterile-filtered and combined with the initial batch of cultured media, and stored at -80°C in aliquots as Wnt3A conditioned medium.

#### Statistical Analysis

The experiments were repeated at least two times. Results are expressed as mean ± SD or SEM as indicated. An independent Student's t-test was performed to analyze the luciferase assay and other analyses. *p *< 0.05 was considered statistically significant.

## Results

### Expression of Twist induces EMT in Hela and MCF7 cells

**Figure 1:**
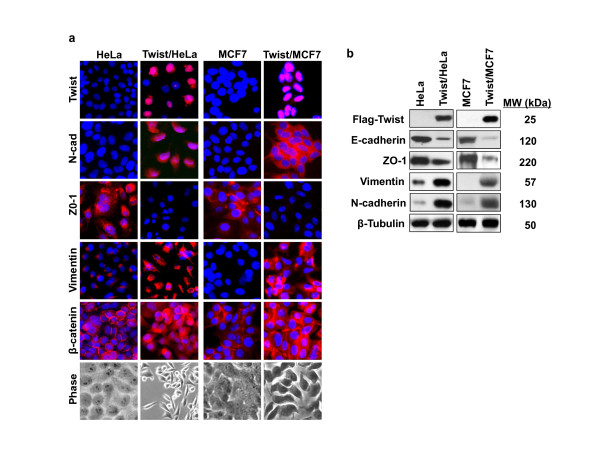
**Overexpression of Twist induces EMT in Hela and MCF7 cells**. (**a**) Hela and MCF7 cells and their corresponding Twist-overexpressing stable transfectants were analyzed for the expression of N-cadherin, Vimentin, ZO-1, and β-catenin by immunofluorescent staining. Nuclei were visualized with DAPI staining (blue). Cell morphological changes associated with EMT are shown in the phase contrast images. (**b**) Expression of E-cadherin, Twist, N-cadherin and vimentin in these cells was analyzed by Western blotting. β-tubulin served as a loading control.

**Figure 2:**
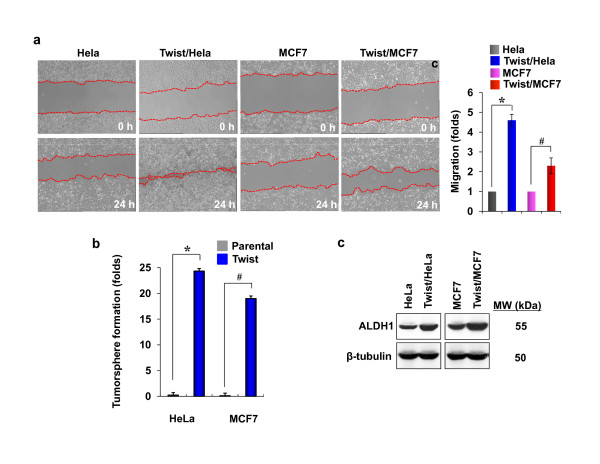
**Twist enhances migration, tumorsphere-formation and ALDH1 activity**. (**a**) Hela and MCF7 cells and their corresponding transfectants were seeded on 6-well plates. After 48 h, a scratch ("wound") was induced in a cell monolayer, and cell culture was continued for an additional 24 h. Images were obtained at the beginning and at the 24 h time point to monitor the cell migration for the closure of the wound. The percentage of cell migration was calculated based on the migration of Hela and MCF7 cells (mean ± SEM in three separate experiments) and the representative images were shown in the left panel. *P < 0.01 for Hela cells compared with the corresponding Twist-overexpressing cells. ^#^*P *< 0.05 for MCF7 cells compared with the corresponding Twist-overexpressing transfectants. (**b**) The tumorsphere formation was measured in Hela and MCF7 cells and in their corresponding Twist-overexpressing stable transfectants as described in Materials and Methods. The statistical analysis for the tumorsphere formation from three independent experiments in duplicate was calculated and presented. * and ^# ^*P *< 0.01 for Hela and MCF7 cells compared with their corresponding Twist-overexpressing transfectants. (**c**) The expression of ALDH1 in Hela and MCF7 cells and their corresponding Twist-overexpressing stable transfectants were analyzed by Western blotting. β-tubulin served as a loading control.

To examine the role of Twist in EMT induction and the generation of stem-cell like properties, we generated Twist-stable expression clones in cervical cancer Hela and breast cancer MCF7 cells. Expression of Twist induced EMT in these cells as morphological changes from a cobble-stone-like shape to a spindle-like appearance were noted; these cells became elongated in shape and disassociated from their neighboring cells (Figure 1a). Immunofluoresent staining showed the upregulation of mesenchymal markers N-cadherin and vimentin and the downregulation of epithelial markers ZO-1 (Figure 1a). Interestingly, β-catenin was accumulated and translocated into both the cytoplasm and the nucleus. Similar results were further confirmed by Western blotting using specific antibodies against E-cadherin, ZO-1, N-cadherin and vimentin (Figure 1b). Consistent with these molecular changes, cell motility was significantly enhanced in cells expressing Twist than that of parental cells (Figure 2a). These results indicate that expression of Twist can induce EMT in Hela and MCF7 cells, which is accompanied with the downregulation of epithelial markers and upregulation of mesenchymal molecules, and thus, results in the enhancement of cell motility.

### Expression of Twist induces stem-cell like properties in Hela and MCF7 cells

The tumorsphere assay, based on the unique property of stem/progenitor cells to survive and grow in serum-free suspension, was successfully used to establish long-term cultures enriched in stem/progenitor cells from invasive tumor samples. To examine whether the expression of Twist induced stem cell-like properties in Hela and MCF7 cells, we performed a tumorsphere formation assay. Surprisingly, the expression of Twist induced about a 24- and 18-fold enhancement in tumorsphere-formation in Hela and MCF7 cells, respectively, compared with that of parental cells (Figure 2b). To further confirm these findings, we also measured the level of aldehyde dehydrogenase 1 (ALDH1), a detoxifying enzyme responsible for the oxidation of retinol to retinoic acid and which has a role in the early differentiation of stem cells. High ALDH1 activity is associated with several types of murine and human hematopoietic and neural stem/progenitor cells. As shown in Figure 2c, the expression of Twist significantly induced the level of ALDH1 in Hela and MCF7 cells.

**Figure 3:**
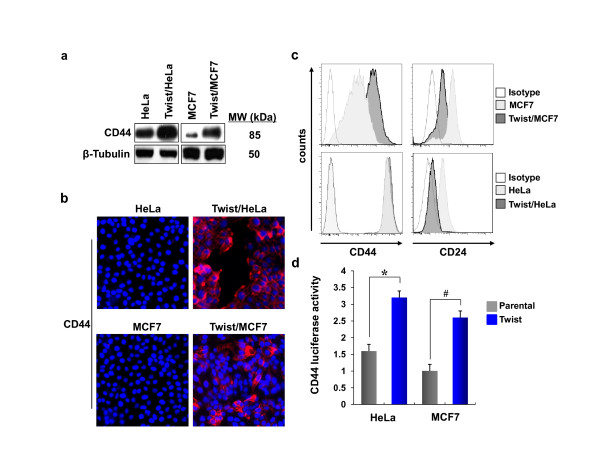
**Twist induces the expression of CD44**. (**a-c**) The expression of CD44 in Hela and MCF7 cells and their corresponding Twist-overexpressing stable transfectants were analyzed by Western blotting (**a**), immunofluorescent staining (**b**), and FACS (**c**). (**d**) CD44 promoter luciferase plasmid was expressed in Hela and MCF7 cells and their corresponding Twist-overexpressing stable transfectants. After 48 hr, luciferase activity was measured by using a Dual-Luciferase Reporter Assay (Promega) (mean ± SD of three separate experiments). * and ^# ^*P *< 0.05 for Hela and MCF7 cells compared with their corresponding Twist-overexpressing cells.

The CD44^high^/CD24^low ^phenotype has been used to isolate stem cells from the human normal mammary epithelium [2]. It has been shown that as few as 200 of these cells generated tumors in NOD/SCID mice whereas 20,000 cells that did not display this phenotype failed to do so. These cells were able to self-renew, differentiate, and display CSC features. To examine whether expression of Twist induces the expansion of this population of cells, we measured the expression of CD44 by Western blotting, immune-fluorescence staining and FACS analyses. As shown in Figures 3a, b and 3c, expression of Twist dramatically elevated the level of CD44 in Hela and MCF7 cells. Consistent with these observations, when CD44 promoter luciferase plasmid was expressed in these cells, the luciferase activity was significantly elevated in Twist-overexpressing cells than that of parental cells (Figure 3d). Together, these results indicate that the expression of Twist is critical in EMT induction, which confers cells with stem-cell like properties by inducing the expression of CD44 and enhancing tumorsphere formation and ALDH1 activity.

### Expression of Twist induces the activation of β-catenin signaling pathway

**Figure 4:**
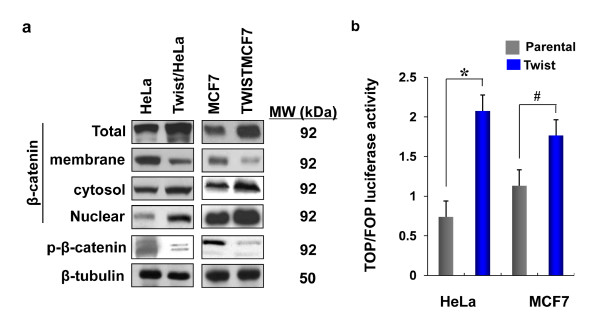
**Twist activates β-catenin pathway**. (**a**) The expression of total, membrane, cytosol, nuclear and phosphorylated forms of β-catenin were examined by Western blotting. β-tubulin served as a loading control. (**b**) The activation of β-catenin was examined by measuring the transcription activity of Top/Fop luciferase in Hela and MCF cells and their corresponding stable transfectants (mean ± SD of three separate experiments). * and ^# ^*P *< 0.05 for Hela and MCF7 cells compared with their corresponding Twist-overexpressing cells.

β-catenin plays an important role in a variety of human tumors. Downregulation of E-cadherin expression often results in an increase of β-catenin, which binds to TCF/LEF to participate in transcription regulation. To test whether the β-catenin pathway was activated in cells expressing Twist, we isolated β-catenin from the membrane, the cytoplasm and the nucleus of parental and Twist-overexpressing cells. Although the membrane-bound β-catenin was significantly decreased, the total level of β-catenin, the cytoplasmic and the nuclear β-catenin were greatly increased in cells expressing Twist (Figure 4a). β-catenin is a labile protein, and it subjected to GSK-3β-mediated phosphorylation and proteasome degradation. Interestingly, we found that the phosphorylation of β-catenin was significantly reduced in cells expressing Twist, suggesting that the increase of the cytoplasmic and the nuclear β-catenin from Twist-overexpressing cells resulted from the release of membrane-fraction β-catenin as well as from the inhibition of phosphorylation and degradation of β-catenin in these cells. To further confirm the activation of the β-catenin pathway, we measured the TOP/FOP luciferase activities. Both Twist-overexpressing cell lines have higher luciferase activities than that of the corresponding parental cells (Figure 4b). Taken together, these data showed that EMT induces an accumulation and nuclear translocation of β-catenin and thus activates the Wnt/β-catenin signaling pathway.

**Figure 5:**
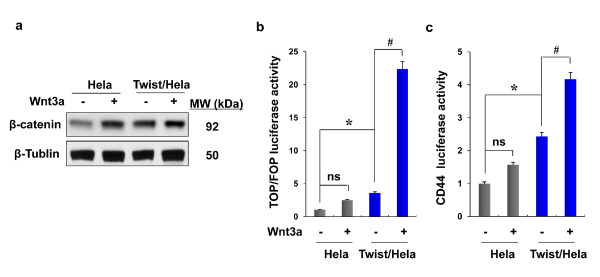
**Wnt3a can further enhance the activation of β-catenin in Twist-overexpressing cells**. (**a**) Expression of β-catenin before or after Wnt3a stimulation (Wnt3a conditioned medium for overnight) was measured by Western blotting. β-tubulin served as a loading control. (**b**) The activation of β-catenin before and after Wnt3a stimulation (Wnt3a-conditioned medium for overnight) was examined by measuring the transcription activity of Top/Fop luciferase in Hela cells and their corresponding stable transfectants (mean ± SD of three separate experiments). *P < 0.05 for Hela cells compared with the corresponding Twist-overexpressing cells. ^#^*P *< 0.01 for Twist/Hela cells compared with cells treated with Wnt3a. Non-significant (ns) was found between Hela cells treat with and without Wnt3a. (**c**) CD44 promoter luciferase plasmid was expressed in Hela cells and their corresponding Twist-overexpressing stable transfectants. After 36 hr, cells were treated overnight with a Wnt3a-conditioned medium and luciferase activity was measured by using a Dual-Luciferase Reporter Assay (Promega) (mean ± SD of three separate experiments). * and ^# ^*P *< 0.05. Non-significant (ns) was found between Hela cells treat with and without Wnt3a.

We also treated Hela cells with Wnt3a, a ligand known to activate the Wnt/β-catenin pathway. As expected, Wnt3a induced β-catenin stabilization in Hela cells (Figure 5a) and a corresponding upregulation of TOP/FOP luciferase activity (Figure 5b). Although Twist-overexpressing Hela cells contained higher levels of β-catenin, and treatment with Wnt3a did not further elevate the level of β-catenin (Figure 5a), Wnt3a can further enhance the TOP/FOP luciferase by more than 10-fold (Figure 5c); this suggests that EMT can synergize the activation of β-catenin induced by Wnt ligands.

CD44 expression was part of a genetic program controlled by the β-catenin/Tcf-4 signaling pathway [26]. Over-expression of the CD44 family is an early event in the colorectal adenoma-carcinoma process, which suggests β-catenin/Tcf-4 signaling is crucial in initiating tumorigenesis [27]. Masaki *et al *supported this result with the immunostaining of β-catenin and CD44, suggesting that the up-regulation of CD44 through nuclear β-catenin contributed to the formation of the tumor [28]. Thus, we measured the CD44 luciferase in Twist-overexpressing cells stimulated with Wnt3a. We found that CD44 luciferase levels were further elevated by Wnt3a (Figure 5c), indicating that the activation of the β-catenin pathway plays a critical role in the expansion of CD44^+ ^cells with stem-cell like properties.

### Expression of Twist activates Akt signaling pathway and increases the level of Snail

**Figure 6:**
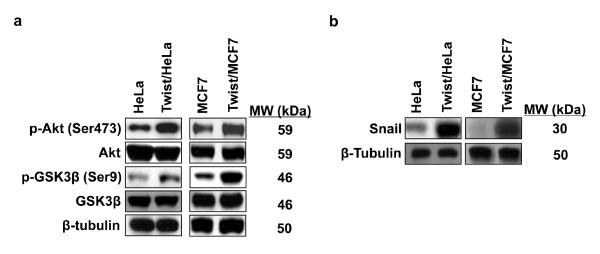
**Twist induces the activation of Akt and the protein stabilization of Snail**. (**a**) The activation of Akt (phosphorylated Akt) and the suppression of GSK-3β (phosphorylated GSK-3β) were examined by Western blotting in Hela and MCF cells and their corresponding stable transfectants. (**b**) The level of Snail in Hela and MCF cells and their corresponding stable transfectants was examined by Western blotting. β-tubulin served as a loading control.

Twist has been shown to activate the Akt signaling pathway by inducing the expression of Akt [29]. To examine whether the expression of Twist activates the Akt signaling, we measured the phosphorylation of Akt in cells expressing Twist and their corresponding parental cells. We found that Akt was activated in Hela and MCF7 cells expressing Twist (Figure 6a). Serine/threonine protein kinase GSK-3β, a downstream target of PI3K/Akt, was also found to be inactivated by phosphorylation at serine 9, whereas the total GSK-3β level remained changed. As GSK-3β can phosphorylate β-catenin and result in its proteasome degradation, this result was consistent with our finding that β-catenin was stabilized because of the significantly reduced level of phosphorylation (Figure 4a). The activation of Akt and suppression of GSK-3β in Twist-expressing cells were quite interesting, as we showed previously that GSK-3β is the major kinase regulating the protein stability and the cellular localization of Snail [30]. To further extend this finding, we examined the expression of Snail in these cells. We found that the level of Snail was significantly higher in Twist-overexpressing cells than that of parental cells (Figure 6b). Together, our results indicate that expression of Twist can induce the activation of Akt and the suppression of GSK-3β, which results in the stabilization of β-catenin and Snail in Hela and MCF7 cells.

### Inhibition of β-catenin and Akt signaling pathways suppress CD44 expression

**Figure 7:**
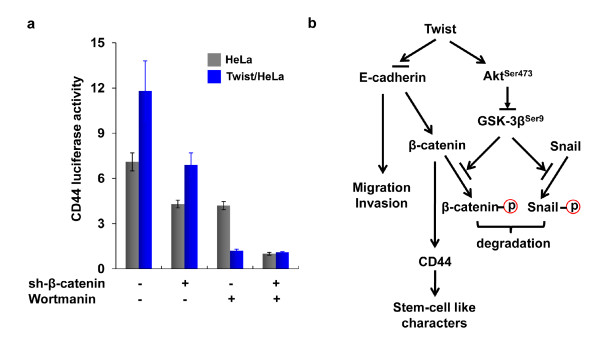
**Blockage of β-catenin and Akt pathways suppresses CD44 expression**. (**a**) The expression of β-catenin was knocked down or cells were treated with wortmannin as described in the Materials and Methods. The luciferase activity of CD44 was measured by using a Dual-Luciferase Reporter Assay (Promega) (mean ± SD of three separate experiments). (**b**) A proposed model to illustrate how Twist activates β-catenin and Akt pathways to maintain the expression of CD44 and stem cell-like properties associated with EMT.

We showed that EMT induced the downregulation of E-cadherin and the detachment of β-catenin from membrane localization. We further showed that EMT activated Akt and suppressed the function of GSK-3β, which is required for the stabilization and nuclear translocation of β-catenin, and thus results in the transcription of CD44. To investigate whether the β-catenin and Akt pathways were critical for the induction of CD44, we knocked down the expression of β-catenin or inhibited the Akt pathway by wortmannin in cells. We found that either the knockdown of β-catenin expression or the inhibition of Akt pathway suppressed the expression of CD44 (Figure 7a). Inhibition of both pathways can further synergistically suppress the expression of CD44, suggesting that the activation of these two pathways is critical for the maintenance of CD44 expression.

## Discussion

In this study, we showed that the expression of Twist induced EMT in Hela and MCF7 cells, and that accompanied the increased stem cell-like properties and the upregulation of CD44. We found that the upregulation of CD44 was mediated by the activation of β-catenin and Akt pathways in these cells; inhibition of both pathways synergistically suppressed the upregulation of CD44. Our study provides several new insights into the regulation of EMT and cell differentiation program. First, our results indicate that the activation of β-catenin and Akt pathways is critical for the maintenance of the stem cell-like properties associated with EMT (Figure 7b). The gain-of-function of stem cell-like properties in EMT may confer tumor cells the survivability against chemo- and endocrine therapies, in addition to a distinct advantage for invasion and metastasis [13,14,19]. However, the molecular link between EMT and the gain of CSCs properties is unclear; whether a shared signaling pathway regulates both processes remains to be determined. The Wnt/β-catenin pathway mediates a wide variety of processes, including cell proliferation, migration, differentiation, adhesion and apoptosis. It is critical for homeostatic stem cell renewal. For example, Wnt signaling is necessary for maintenance of stem cells in the intestinal crypts [31]. Treating prostate cancer cells with stem cell-like characteristics with WNT inhibitors reduced both the size of tumorspheres and the ability of self-renewal, whereas Wnt3a stimulates them [32]. Consistent with previous reports [12-15], we found that over-expression of Twist induced EMT in Hela and MCF7 cells, which accompanied the gain-of-function of stem cell-like properties, such as high levels of ALDH1 expression, tumorsphere-formation and high levels of CD44. We further showed that the β-catenin pathway was activated as the membrane-bound and phosphorylated β-catenin was significantly decreased in Twist-overexpressing Hela and MCF7 cells. E-cadherin is known to anchor and to sequester β-catenin in the membrane and prevent it from activation; the activation of β-catenin signaling may result from the downregulation of E-cadherin at EMT. CD44 has been shown to be a downstream target of the β-catenin signaling pathway. We found that elevated CD44 correlated with the activation of β-catenin in Twist-overexpressing cells. Interestingly, the activation of the β-catenin pathway was not optimal, as treatment of Wnt3a can further induce the activation of β-catenin and the induction of CD44, suggesting that EMT initiates and primes β-catenin activation and this activation can be further synergized by the Wnt ligand from the tumor microenvironment.

The expression of Twist also has been shown to activate the Akt pathway to promote migration, invasion and paclitaxel resistance [29]. The activation of Akt phosphorylated and suppressed GSK-3β, which is the major kinase for the phosphorylation of β-catenin and Snail [30,33]. The phosphorylation of these molecules by GSK-3β results in the consequent degradation of β-catenin and Snail by E3 ligase β-Trcp [30,33]. Consistent with these findings, we discovered that Akt was activated in Twist-overexpressing cells, which lead to the phosphorylation and suppression of GSK-3β and resulted in the significant protein stabilization of β-catenin and Snail in these cells. When E-cadherin is downregulated at EMT, the released cytoplasmic β-catenin is still subjected to GSK-3β mediated phosphorylaton and degradation. Thus, additional activation of the Akt pathway is necessary to prevent this process and facilitates the nuclear translocation and activation of β-catenin. This speculation is consistent with the fact that EMT also correlates with the presence of β-catenin in the nucleus [34]. Thus, activation of β-catenin and Akt pathways is a synergistic event at EMT and is critical for generating high-grade invasive cells with stem cell-like features (Figure 7b).

Second, our results suggest that targeting the β-catenin and Akt pathways can suppress the stem cell-like properties associated with EMT. CSCs are often resistant to common drugs *in vivo *and *in vitro *when compared with the majority of the cancer cell population, raising the question of whether traditional therapy only "debulks" tumors, leaving CSCs to repopulate the original tumor and which results in disease recurrence. Consistent with these findings, Cheng and her colleagues showed that the residual breast tumor cell populations that survived after conventional treatment were enriched for the subpopulation of cells with both tumor stem cell-like features and EMT characteristics [9,11]. Thus, more effective therapies will require the selective targeting of this crucial cell population. The elucidation of molecular pathways underlying the regulation of CSC self-renewal and survival is crucial to the success of this goal. In our study, we found that either the knockdown of β-catenin expression or the suppression of the Akt pathway by wortmannin inhibited CD44 expression. Moreover, the combination of both chemical suppression and siRNA knockdown significantly suppressed the expression of CD44, indicating the synergistic effect of these two pathways in maintaining the stem cell-like properties associated with EMT. Gupta et al. recently implemented a chemical screen and discovered compounds showing selective toxicity for breast CSCs, including salinomycin [35]. It would be interesting to test whether Salinomycin inhibits the activation of β-catenin and Akt pathways in the near future.

## Conclusion

In summary, we showed that the activation of β-catenin and Akt is critical for the maintenance of CD44 expression associated with EMT. Targeting these pathways, in conjunction with currently used conventional treatments, may provide a new therapeutic strategy for eliminating surviving tumor cells to prevent recurrence and to improve long-term survival in cancer patients.

## List of abbreviations

ALDH1: aldehyde dehydrogenase 1; APC: adenomatous polyposis coli; CKI: casein kinase I; CSC: cancer stem cell; EMT: epithelial-mesenchymal transition; FACS: Fluorescence-activated cell sorting; GSK-3β: glycogen synthase kinase 3-beta; TCF: T cell factor

## Competing interests

The authors declare that they have no competing interests.

## Authors' contributions

JL participated in the design of the experiments, performed experiments and wrote the initial draft of the manuscript. BPZ designed experiments, interpreted results and wrote the final draft of the manuscript. Authors read and approved the final manuscript.

## Pre-publication history

The pre-publication history for this paper can be accessed here:

http://www.biomedcentral.com/1471-2407/11/49/prepub
